# Veterinary decision making in relation to metritis - a qualitative approach to understand the background for variation and bias in veterinary medical records

**DOI:** 10.1186/1751-0147-51-36

**Published:** 2009-08-30

**Authors:** Dorte B Lastein, Mette Vaarst, Carsten Enevoldsen

**Affiliations:** 1 Department of Large Animal Sciences, Faculty of Life Sciences, University of Copenhagen, Grønnegårdsvej 2, DK-1870 Frederiksberg C, Denmark; 2 Department of Animal Health, Welfare and Nutrition, Faculty of Agricultural Sciences, Research Centre Foulum, University of Aarhus, P.O. 50, DK-8830 Tjele, Denmark

## Abstract

**Background:**

Results of analyses based on veterinary records of animal disease may be prone to variation and bias, because data collection for these registers relies on different observers in different settings as well as different treatment criteria. Understanding the human influence on data collection and the decisions related to this process may help veterinary and agricultural scientists motivate observers (veterinarians and farmers) to work more systematically, which may improve data quality. This study investigates qualitative relations between two types of records: 1) 'diagnostic data' as recordings of metritis scores and 2) 'intervention data' as recordings of medical treatment for metritis and the potential influence on quality of the data.

**Methods:**

The study is based on observations in veterinary dairy practice combined with semi-structured research interviews of veterinarians working within a herd health concept where metritis diagnosis was described in detail. The observations and interviews were analysed by qualitative research methods to describe differences in the veterinarians' perceptions of metritis diagnosis (scores) and their own decisions related to diagnosis, treatment, and recording.

**Results:**

The analysis demonstrates how data quality can be affected during the diagnostic procedures, as interaction occurs between diagnostics and decisions about medical treatments. Important findings were when scores lacked consistency within and between observers (variation) and when scores were adjusted to the treatment decision already made by the veterinarian (bias). The study further demonstrates that veterinarians made their decisions at 3 different levels of focus (cow, farm, population). Data quality was influenced by the veterinarians' perceptions of collection procedures, decision making and their different motivations to collect data systematically.

**Conclusion:**

Both variation and bias were introduced into the data because of veterinarians' different perceptions of and motivations for decision making. Acknowledgement of these findings by researchers, educational institutions and veterinarians in practice may stimulate an effort to improve the quality of field data, as well as raise awareness about the importance of including knowledge about human perceptions when interpreting studies based on field data. Both recognitions may increase the usefulness of both within-herd and between-herd epidemiological analyses.

## Background

Files with information on animal disease have a variety of applications at both the herd and national level, including monitoring the incidence of animal diseases or medical treatments, analyses of causal relationships, bench marking, estimation of treatment criteria, effectiveness of treatment on production, etc. Such information necessarily must be gathered from multiple observers in a wide range of contexts (e.g., the Danish national cattle database). Both disease detection and criteria for treatment are influenced by human perception, as exemplified by a study of farmers and mastitis [1]. This influence introduces the possibility of both variation and bias (e.g., problems related to intra- and inter-observer agreement). Consequently, consideration of data quality in existing data files becomes essential before any quantitative analysis can be conducted and interpreted. Intra- and inter-observer agreement about the manifestations and criteria for treatment must be estimated (quality control), because different people often judge the same conditions differently, as discussed by Baadsgaard and Jorgensen [2].

Disease manifestations or 'diagnostic data'--e.g., which clinical signs of metritis can be seen or scored--should be clearly distinguished from treatment records or 'intervention data'. In the Danish Central Cattle Data Base, it is now possible to record information about disease--for example, as various types of scores--and medical treatments separately. This option is primarily used in case of metritis in dairy cows in herds participating in a recently implemented herd health programme [3]. The metritis diagnosis is recorded as an ordinal score with values from 0 to 9 (higher score corresponds to a more 'severe' disease). The scores are gathered by veterinarians between 5 and 21 days in milk from all cows calving in the herds. Medical treatments of metritis are also recorded by the practicing veterinarians, because farmers' use of antibiotics is restricted by Danish legislation.

In summary, the individual veterinarian records two distinct variables: 1) Diagnosis, that is, a score based on observed clinical signs of metritis, and 2) Treatment decision, that is, determining treatment or non-treatment based on criteria for treatment classification. The consequence is that disease incidence can be described separately from disease treatment incidence.

In this article, data collection related to metritis in dairy cattle is investigated empirically and is discussed as an example of problems that must be addressed prior to and during quantitative analyses of such data. The aim of the study is to explore qualitative aspects and potential mutual influences of collecting metritis score data and metritis treatment data, and how the relationship between these two types of data is influenced by human perceptions and decisions. The study also considers potential consequences for the quality and subsequent analysis of field data on herd and national levels. The research tool is qualitative analysis of observations in veterinary practice and statements from semi-structured interviews.

## Methods

### The context

Legislation for a new type of voluntary dairy herd health programme was introduced in Denmark in 2006 [3]. The programme aims at improving the detection and registration of the most important health disorders to allow accurate monitoring of the development of disease incidence over time, hence using these data for disease control measures. The veterinarian and the farmer join the programme by signing a 'herd agreement' specifying a set of rules for mandatory systematic data collection. This agreement gives the farmer a more liberal access to antibiotics. The intention behind this legislation probably was to motivate the farmer to enhance disease prevention through dialogue with and the advice given by the veterinarian. By the end of 2008, approximately 100,000 cows, or approximately 20% of the total Danish dairy cattle population, were enrolled in the program. In these herds, all treatments and scores related to metritis must be recorded systematically, according to a common manual (consult table 1 to see the scorings of metritis) and entered into the Danish Central Cattle Data Base.

**Table 1:** Table of metritis score definitions and examples of present usage in practice.

**Scores**	**Clinical signs - vaginal examination**	**Cases**	
		
		***Practical scoring***	***Decision making on treatment***
0	None or very small amount of clean mucous discharge - no odour	L elaborates on the use of score 0: "Well, some should maybe have been 1 or 2. The score 1 I have never used." L scores all cows with a normal puerperal discharge 0.	
		
1	A very small amount of bloody mucous discharge - no odour		
		
2	Small amount of bloody mucous/grey discharge - no odour		
		
3	Large amounts of bloody seromucous/grey-yellow discharge - scabs on tail - no odour	J: "I use 2 - which means I will not treat, but I would like to see the cow again for control [...] I could use 3-4. But I just use 2, and the farmer knows what it means". J uses 0 for cows that are immediately characterized as non metritic.	

4	Large amounts of grey/yellow seromucous discharge - no abnormal odour	K: "My metritis score 4. It is when there is plenty of discharge, that smells and there is no temperature". J: "I can not differentiate as sharp as it is suggested by the system, so I only use 5-7-9".	A uses 4 and rectal temperature as a minimum threshold for metritis treatment.
		
5	Little to medium amounts of purulent discharge - difference in consistency and colour - smell abnormal		L uses the combination of score 4 and a flaccid uterus by rectal examination to initiate treatment with prostaglandin.
		
6	Medium amounts of discharge - difference in texture and colour - smell abnormal		K, I, E, J & B are explicitly using 5 as a minimum threshold for treatment.

7	Medium to large amounts of discharge - beginning to look red-brownish - stinks	I: "I have never given a cow score 9 if she was not very ill. We saw a cow I gave 8 [...]If she had had sunken eyes I had probably given her 9 with the same vaginal findings"	D, C, L, & H using a variable threshold for treatment and makes individual decision on individual cows based on multiple clinical criteria (incl. metritis score).
		
8	Large amounts of greyish discharge - stinks	K's scoring is influenced by rectal temperature: the higher temperature, the higher metritis score.	H attempts to exclude score 8-9 from the scale: "If they have a cow there is as sick as 8-9 they should call in advance. "
		
9	Large amounts of brown-yellow/brown discharge- typically a retained placenta - "smells like h...!"		

The table explains the metritis scores with definitions. Cases from the interviews are given to demonstrate how the scores are used in a practice context, and how they are used during decision making for determining treatment threshold for metritis. Capital letters refer to specific veterinarians.

The programme is based on systematic weekly/fortnightly clinical screening of all cows in a herd at specific expected high disease risk periods, i.e., at drying off and at calving (5-21 days *post partum*). The mandatory screenings focus on general condition, metritis/vaginitis, mastitis and body condition. Optional screenings focus on ketosis and limb disorders [3]. No official treatment threshold was linked to the metritis scale, but leading Danish veterinarians in the field recommend using a grade of 5 on the scale as a cut-off value for initiating medical treatment, and statements from veterinarians at meetings indicate that this criterion seems to have been generally accepted as a rule of thumb.

### Selection of participants

A list of veterinarians with two or more 'herd agreements' within 3 geographical regions was obtained from a central registry of veterinarians. Veterinarians were phoned, starting at the top of the list. Twelve veterinarians, with between 2 and 15 herd agreements per veterinarian; (median: 4 herds) and with from 3 to 30 years of experience in cattle practice agreed to participate after a short introduction. Only one veterinarian from each practice was included. Anonymity was guarantied to promote openness and confidentiality.

### Participant observation

Observations of veterinary work on farms and interviews were made by the first author [DBL] from January to March 2008. The veterinarians were observed during 1-4 herd visits when the veterinarian did practical scoring and medical treatments. Observations and discussion notes from the herd visits were used later to initiate and guide the interviews of the veterinarians.

### Qualitative semi-structured research interviews

All veterinarians were interviewed about their decisions related to metritis using a semi-structured research methodology [4]. The duration was 1/2 hour to 1 1/4 hour per interview. Based on the observations, cases, herd documents and interview themes (table 2), the veterinarians were encouraged to tell about their own personal experiences, perceptions and practical observations regarding diagnosis (including scoring) and treatment of metritis. DBL directed the conversation through the themes and followed-up on the statements given by the interviewed veterinarian. Most interviews were initiated by either a general opening: 'Could you comment on your thoughts on metritis treatments in the scheme' or more specific: 'This morning I [DBL] observed the following situations in a herd (e.g. scoring a cow and initiating a metritis treatment), would you please elaborate on that specific situation?'

**Table 2:** Interview themes

Clinical registration
Diagnostic criteria
Treatment strategies
Treatment effect in relation to production parameters
Control of clinical effect
Herd status
Farmer's influence
Influence of strategy in veterinary practice
Ideology
Legislation

### Data Analysis

The qualitative analysis is based on a phenomenographic approach; that is a qualitative method to use empiric data (e.g., interview) to describe the variation in and logical relations between human perceptions of a phenomenon [5,6]. All interviews were recorded with a digital voice recorder and transcribed in full length. Different forms of interaction between practical metritis scoring and treatment decisions were identified. Statements or parts of the interview with a coherent meaning were condensed into short, descriptive headings in a process called 'meaning condensation' [4] Headings were categorized, as we identified differences in the way veterinarians experience the phenomenon of generating score data and decision making in relation to treatment of metritis and their motivation to produce data. This information was condensed into a 'model of understanding' that demonstrates the relationship between perceptions and data quality. The veterinarians' perceptions of the reasoning behind their own decisions were explored. Citations are typically used to demonstrate typical views and meanings.

## Results

### The use of metritis scores for decision making

**Figure 1:**
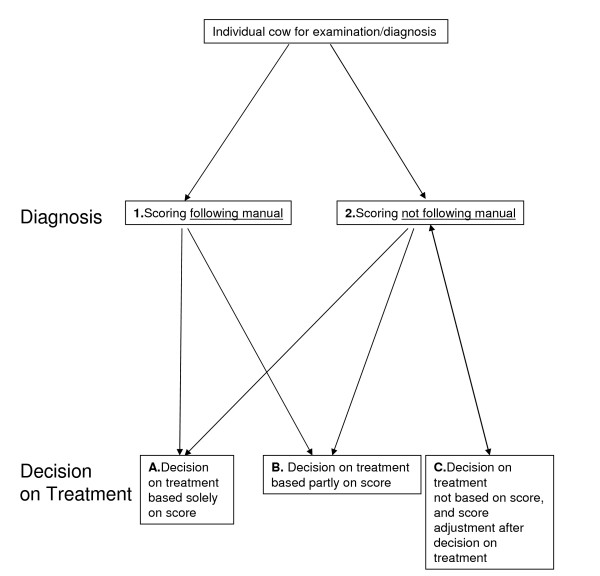
**The interactions between diagnostics (incl. metritis score) and decisions on treatment of metritis**. The diagram shows that for individual cows diagnosed with metritis, several different pathways of decision related to the metritis score are taken by the interviewed veterinarians.

All veterinarians initially stated that they used the metritis score as a means to identify a need for treatment. In Table 1, cases of the practical use of metritis scores and decision making on treatment are described. These cases exemplify that the practical usage involves implicit adjustments of treatment criteria to a given situation, i.e., explicit criteria of treatment are not necessarily used by the individual veterinarian. Three types of interactions between scoring and decisions of treatment were identified (Figure 1).

As illustrated in Figure 1, one category of veterinarians based their treatment decisions entirely on the metritis score (case 1). Another category of veterinarians included other observations in the treatment decision (case 2). One example also demonstrates how the metritis score was manipulated in order to fit the decision already taken by the veterinarian concerned, but was based on other implicit (not recorded) observations (case 3).

**Case 1**. In the interview we touch upon organic farmers' explicit wish to minimise the use of medicine, either because of ideology, association between treatments and longer withdrawal period of milk in organic herds, or for other reasons. As an aid to understanding the quote, note that the veterinarian equates 'smell' and metritis score 5 or higher, and that legislation requires that follow-up treatments are done by veterinarians in organic herds.

DBL: "I was wondering if you are running this programme in an organic herd - and the farmer argues for minimal medicine usage - for both economic and ideological reasons. Would you change your treatment threshold?"

VETERINARIAN:" Not voluntarily! I will always treat the ones that smell. Perhaps I could reduce the length of treatment, if the farmer is cranky about it; also because we have to do the follow-up treatment ourselves. Otherwise I always treat a minimum of two days after first treatment."

**Case 2**. The case is based on an observation in a herd, where DBL had observed the veterinarian examining a cow and recorded a metritis score of 7. The veterinarian decided not to treat the cow. He was asked to elaborate on the case:

VETERINARIAN: "It's a question about looking at the cow. It did not have fever, and it looked 'nice'. No reaction on ketosis sticks. So a score 7 - I believe that the cow can manage the disease without treatment, because she has a good general condition. Treatment might be an issue later - perhaps only because of sequels for reproduction. But my immediate appraisal is that the cow requires no treatment."

**Case 3**. The treatment criteria were discussed with the veterinarian in case 3. The veterinarian that had selected a treatment criterion at score value 5 had told DBL during the morning's herd visits that 'a cow scored 5 could smell more in one herd than in another'. He is asked to elaborate on the statement during the interview.

VETERINARIAN: "When you stand with your hand in the cow without knowing whether you should treat or not, then I look at the cow; body condition score, milk yield, rectal temperature - and which herd she is in. The herd management means a lot. In some herds she may be left in a corner, and maybe ... what if her metritic condition worsens? In these herds I treat the cow. In other herds she will never be overlooked. In other herds it is absolutely certain that they'll call me in two days if the metritis condition develops."

DBL: "Do you then score 4 in 'herds where you do not treat'?"

VETERINARIAN: "Yes - because a 5 is treated. The score 5 will vary between herds, but only a little bit."

### Model of understanding with regard to decision levels

**Figure 2:**
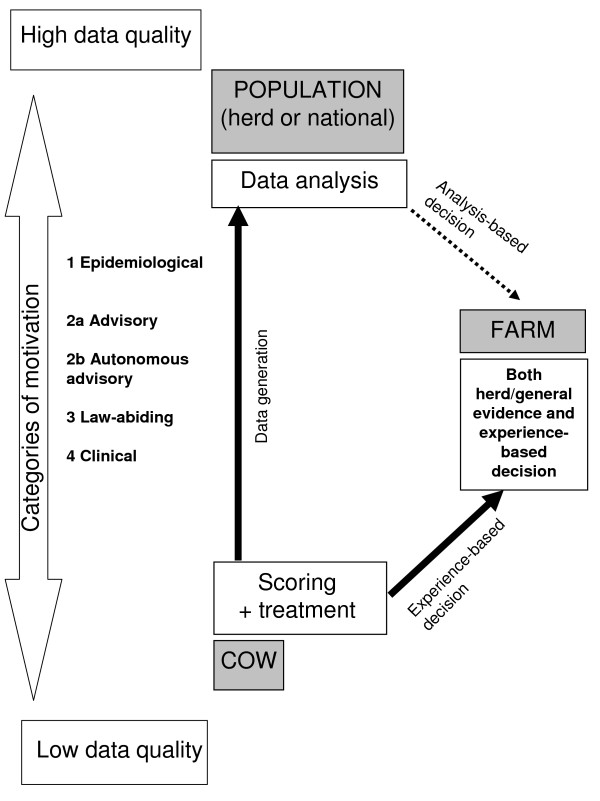
**Model of decision levels and categories for motivation**. The model shows that veterinarians work on the cow, farm or population level. They generate data between the cow level (scoring and treating metritis) and the population level (data analysis), and potentially use observation or data through either experience- or evidence-based decisions at the farm level. Quality of data (e.g., intra and inter observer agreement) is affected by the 'categories of motivation'. Consequently, the data are more or less suited for subsequent analysis-based decision making on farm and population level. The dotted arrow between population level and farm level indicate that few veterinarians use data analysis in their daily practice and advice.

Based on analysis of the veterinarians' perceptions of how they wished to use the metritis score in their practice and on dialogue with the farmer and surroundings in general, a model of understanding was developed (Figure 2). Three levels of decision were revealed: cow level (individual cows), farm level (multiple cows in a specific farm) and population level (multiple cows in multiple farms). None of the veterinarians took decisions exclusively on one level or were motivated solely through one category of motivation, but they might have been more or less focussed on each of the three levels/categories of motivation.

At the level of the individual cow, the veterinarians seemed to base their treatment decisions on the cow's characteristics. They focussed generally on the practical use of the score to support treatment of each individual cow, indicating that decisions can differ both within and between herds.

At the farm level, the veterinarians seemed to integrate farm-related information into the decision as to how to treat an individual cow for metritis. When taking decisions on this level, a veterinarian often used predefined herd-specific standard treatments, sometimes with considerable variation between herds (e.g., milk withdrawal period due to individual farmers' wishes). To various degrees, the veterinarians included practical conditions and perceptions such as farmers' inability to manage follow-up treatments or restrain cow properly for intravenous injection. This can give a pattern of treatments which is strongly influenced by the veterinarian's perception of the specific farm and by his or her evaluation of the local context. That is, treatment data as an indicator of a certain disease manifestation may only be valid within the herd.

When veterinarians used standard treatment decisions and included population level considerations and general evidence into the criteria (e.g. using the same cut-off value on metritis scale in all herds), they were generally focussed on the importance of generating data for valid epidemiological analyses across herds. They would therefore both score metritis and make decisions on treatments in a more uniform way across herds, attempting to produce data of both high accuracy within-herd and between-herd.

### Categories of motivation for generating data

Four different categories of motivation among the veterinarians for collection and usage of the metritis data were derived from the analysis and given the headings: 1) epidemiological, 2a) advisory, 2b) autonomous advisory, 3) law-abiding and 4) clinical. In Figure 2, the order of these categories is based on the authors' suggestion concerning how these motivations may link to the decision levels and, consequently, data quality. Each veterinarian could be influenced by different motivational factors as described above.

#### 1) Epidemiological

Veterinarians motivated by epidemiological considerations would follow the guidelines for the scoring and would treat based on certain criteria which vary little between cows and herds, so as to be able to create meaningful data valid in large scale analyses (across herds and veterinary practices). Such veterinarians would generally want to focus on possibilities for across-herd data analyses and, with time, be able to formulate meaningful disease control strategies based on empirical data at the herd level. Veterinarians in this category are aware of the possibility of actually basing their decisions on epidemiological analyses in the future, and they are highly motivated to use, for instance, multi-factorial analysis on the herd level or higher levels in their daily work (Figure 2).

#### 2a) Advisory

Veterinarians could be motivated by the capacity of scores to function as an entrance to advisory services on the farm level. Such veterinarians are motivated to collect valid data at the herd level. They perceive the collection of the data in and of itself as the basis for taking relevant action at the farm. They may skip the process of systematic analysis of data and give advice based on their immediate evaluation of the results compared to previously collected data ('qualitative monitoring'). Consequently, they are typically focused on internal validity within each farm context, which may make them less concerned with the problems of adjusting treatment criteria and types of treatments between herds. However, 2 subgroups of advisors are identified, 2a) that follow score definition - making data both valid within herd and potentially valid between herds--and 2b) that act autonomously as described below.

#### 2b) Autonomous advisors

These are veterinarians who primarily followed their own definitions of different scoring values, such as excluding certain scores (see examples in table 1). They find the definitions incorrect. If the veterinarian strictly follows his/her own scoring guidelines, the data will be internally valid, but clearly cannot be used between herds.

Autonomous veterinarians are, in general, motivated by the combination of analysis- and experience-based decisions; they act autonomously in the sense that they appreciate the results of analysis, but only if it becomes integrated into the local herd context.

#### 4) Law-abiding

Veterinarians stated that metritis scoring is enforced by law. This was the primary motivating factor for running the herd health programme, rather than, for example, creating possibilities to perform epidemiological analyses or base advice on systematically collected data. This motivation could potentially lead to 'justifying,' i.e., adjusting of the score to fit to the treatment decision. This category of veterinarians based the treatment decision on an overall evaluation of the case, irrespective of the existence of scores.

#### 5) Clinical

These *v*eterinarians clearly spoke of the scores as a 'diagnostic tool' related to each individual treatment decision rather than being part of a collaborative data collection. For example, they could add rectal temperature and other parameters into the scoring (see Table 1 for examples), which might also lead to lack of data validity, though seen from a clinical point of view, highly relevant. Veterinarians who claimed to be motivated by the use of scoring and data collection for their immediate clinical decisions also included their perceptions of treatment prognoses and experiences from relatively few cases. Veterinarians in this category primarily base decisions about treatment (and/or advice in general) on their personal experience (Figure 2), and not on the basis of analysis, as their 'epidemiological counterparts'.

### External factors influencing treatment decisions

Based on the interviews, we identified four types of influencing factors related to treatment decisions:

1. *Production/economy: *Some veterinarians emphasised the positive influence of timely treatments on production in terms of increased milk yield and improved fertility. This also includes considerations on withdrawal time of milk.

2. *Animal health/welfare: *Some veterinarians claimed to consider this as a driving factor when treating as early as possible. Some interviewed veterinarians also referred to experiences with reduced risk of left displaced abomasum and early cullings due to metritis as result of following this programme.

3. *Common strategies in groups of veterinarians: *Some veterinary group practices had developed common 'good practice treatment strategies' (e.g., application of corticosteroids in addition to the antibiotic treatment), which influenced all decisions of each individual veterinarian, and yet still left room for context specific evaluations and decisions.

4. *Public health/antibiotic resistance: *Concerns related to spread of antimicrobial resistance could lead to the non-use of broad-spectrum antibiotics and intrauterine treatments.

## Discussion

### Considerations on validity of qualitative analysis

Qualitative research methodologies are often used to understand aspects of human perception of life in general and have earlier been used in veterinary sciences for similar purposes [1,7,8]. In this particular interview study, the aim was to reveal perceptions and reasoning behind generation of data and to describe the interaction and relations between the recording of metritis scores and veterinarians' decision making connected to metritis treatment and potential links to data quality. This understanding provides insight into potential errors (bias and random error) related to data based on clinical examinations and human decision making, although it may not cover all possible sources of bias in the whole population of veterinarians.

The study makes use of an inductive research methodology called phenomenography [6] We aim at identifying categories of perception of the phenomena; 'scoring and recording data on metritis' that relate to the quality of the data that are produced. We analyse and build 'a model of understanding' based on DBL's observations and the individual veterinarians' perceptions expressed in their local context. Our basis for the model is thus the empirical data and not an initiating general theory or hypothesis. From these data we wanted to identify a limited number of ways to understand the phenomenon. It was therefore essential to extract as much information as possible from each context of interest, allowing in this case for a long interaction period between each interviewed cattle veterinarian working with a herd health programme and the researcher. In qualitative research, the data collected from each interviewee should be regarded as the sum of words, tone of voice and body expressions observed during the interaction period, as well as the observer's immediate feelings, experiences, and thoughts on the subjects and the observed [9]. However, we acknowledge the risk of influential interaction between the interviewer and the interviewed during the interviews that could influence the statements of the interviewed e.g., the use of leading questions.

In the phase of analysis it is important to determine when no additional information can be extracted from the interviews and field observations or from additional interviews [9]. 'Information redundancy' or 'data saturation' is a measure of the power and validity of the qualitative studies [9]. Information redundancy or data saturation is reached when we are able to build a model that describes the phenomenon coherently with no internal contradictions. There are no exact criteria to determine when that state is attained. The number of participants (12) was chosen in this study and is in accordance with recommendations for this type of research [9]. Detailed discussion on the methodologies including issues of representativeness and validity, and hence the usefulness of data for quantitative and qualitative research, can be found elsewhere [9,10]. However, it is important to emphasize that the methodology and study design do not enable us to make inferences on the number of veterinarians in each identified categories of motivation. That is, we cannot estimate the quantitative distribution of various ways of reasoning or to give quantitative estimates of bias and random error. This will require another study design. The results of the present study could potentially provide the basis for such a study.

### Considerations on data quality and different quantitative analysis

The epidemiological issue of variation and bias are linked tightly with the terms accuracy and precision. Accuracy and precision of disease detection and classification methods at the cow level over time are central to minimizing variation and bias, regardless of the later use of the data for quantitative analyses. Definitions of accuracy and precision here are defined in accordance with Dohoo *et al*. [11]. Accuracy means the average similarity between the observation/classification and the 'true disease state/class'. Because no gold standard for metritis scoring and few validated criteria for metritis treatment exist at present, the accuracy of observations (scores) and classification (treatment or not) cannot be evaluated against a 'gold standard'. However, under the assumption that the 'true disease state/class' exists, observers' ability to score or classify accurately within and between observer and within and between herd will influence the validity of data, and hence the subsequent analytical use either within the herd ('herd analysis') or between herds ('national analysis'). Accuracy within observer is a prerequisite for valid 'herd analysis' (assuming one observer per herd), and accuracy between observers is a prerequisite for valid 'national analysis'. Precision means the similarity between multiple scorings or classifications of the same condition, either within or between observers. In practice the same cow will very rarely be evaluated twice by the same or another observer at the same time point. In any case, the importance of precision seen from an analytical point of view relates to number of observations required to reveal non-random differences between groups ('significance testing'). Hence sources of variation and bias (poor accuracy and precision) in centrally collected data files--including unstructured human influence--must be revealed, evaluated and discussed in depth prior to a quantitative analysis. This may allow subsequent analytical control of bias.

### Sources of bias and variation in veterinary records

Records of metritis scores, ideal for monitoring of disease incidence, should not be influenced by metritis treatment data, because the scores should be given on the basis of strictly defined criteria and should be calibrated within and between observers. Neither should the metritis score be influenced by factors which could potentially influence a treatment decision (e.g., recorded daily milk yield). The treatment data, ideal for epidemiological analysis, should be a result of validated known (explicit) treatment criteria to ensure comparability between cases/non cases, while registrations of additional explicit factors should provide a basis for analytical control of interactions and confounding. However, central data bases are based on field data from multiple observers, which create non-ideal data. In practice, treatment decisions often involve a complex set of observations based on previous experience, local context and external evidence, a situation similar to the concept of evidence based medicine [12].

We have shown in accordance with Kristensen *et al*. [7] that lack of uniformity of scores (e.g., different scores within the same clinical entity and adjustment of scores to suit decisions) leading to reduced intra- and inter-observer agreement are likely to occur in medical records of field data. The sources of misclassification bias (e.g., differences in treatment criteria for metritis scores within and between herds) can represent both the lack of clear case definitions in field data and the use of different opinions on when to treat, also in cases where different observers might agree on the metritis score they use (case 1 versus case 2 - fixed versus varying criteria for treatment). Further, we have identified interaction and feedback mechanisms between diagnostic observations (scores) and decisions (criteria to treat) which implicate that errors are not independent. Some veterinarians regard the two records as totally correlated, others regard them as entirely independent, and still others regard them as correlated, but adjust the score to suit a decision taken (justification).

This study indicates that some veterinarians working within the herd health programme are primarily focused on case-related problems (at the level of the individual cow), hence lack focus on potential subsequent use and validity of their clinical records in a broader perspective. On basis of this, we suggest that the importance of the epidemiological aspects on data quality of field data should be articulated and emphasised in the education of veterinarians, both at student and post-graduate level.

### Potential consequences of bias and variation in veterinary records

Veterinary medical records can be applied in the dairy sector in many ways and for many reasons. In the following we will discuss the consequences of variation and bias in relation to monitoring of animal disease incidence on herd and national level, causal analysis on national level, as well as estimation of validated treatment criteria.

Monitoring of disease incidence (metritis score) over time can be used on the herd level to evaluate, for instance, effects of preventive interventions. Observers within the same herd should be able to obtain unbiased data. Accuracy between herds is irrelevant for evaluating data on herd level e.g. over time. Improved precision of the scores (less variation) will reduce the number of observations needed to obtain an acceptable level of certainty. If metritis is monitored as part of a national programme, accuracy between veterinarians is required. The large variation in the use of the metritis scores and treatment criteria between veterinarians revealed in this study indicate that there is a huge variation between observers. This should clearly be improved before analysing the data on national level.

Causal analysis of cow-level and herd-level risk factors for metritis at the national level based on Danish central data base files was performed by Bruun *et al*. [13] using treatment data as measure of disease. Our study shows that it is very difficult to give a valid biological interpretation of results from across-herd estimates of quantitative associations between clinical conditions (e.g., metritis scores) and disease treatments. The statement from case 2, above -'*I believe that the cow due to a good general condition can manage the disease without treatment' *- demonstrates that such associations are influenced by multiple factors, both explicit (e.g., acceptable milk yield) and implicit (e.g., perception of good prognosis). This particular veterinarian in case 2 chose to not treat a cow despite a metritis score of 7 (stinking discharge - see table 1 for detailed description). This veterinarian's perception of 'good condition' (true or not) might lead to a lower probability of treatment in average to high yielding cows.

Treatment criteria can be discussed and to some extent calibrated between veterinarians. This would improve comparability between cases and non-cases from different settings, and enable researchers to take into account additional variables in subsequent analyses.

Our study shows that variation and bias in field data (records of metritis scores and metritis treatment) within the herd health scheme are very likely and that the origin is complex, sometimes including feedback. When regularly trained and calibrated, the group of epidemiologically oriented veterinarians might provide data on the metritis scores that are valid for subsequent across-herd analyses of, for instance, quantitative relations between metritis and risk factors or effects of metritis on production. The problem will be to identify the veterinarians belonging to this category in a large file with routinely collected data.

The association between (true) disease state and treatment probably cannot be detected and recorded systematically in all herds, especially not when treatment criteria are based on a combination of factors and rarely made explicit. Consequently, analytical control is probably not possible. If the implicit and explicit treatment criteria are applied on a larger scale, underestimation of effects may occur in some herds, overestimation in others. Unfortunately, there seems to be little evidence in across-herd studies that this problem is even recognized in depth and dealt with. The feedback mechanisms between outcome and risk factor, as well as the interaction between risk factor and herd/veterinarian revealed in this study suggest that observational studies, including meta-analysis, should be interpreted with caution. Including 'random effects' of herd or veterinarian in the analyses will not solve all the problems revealed in this study (e.g. feedback and interaction).

Results of randomised clinical trials can supplement studies involving observational data by creating an understanding of connections between clinical signs and treatment criteria. Only a few controlled clinical trials on early metritis diagnostics and treatments are published. Consequently, little 'external evidence' can be found in the literature concerning diagnosis and treatment of 'early metritis' [14-17]. This means that very little guidance based on epidemiological analyses or systematically collected veterinary experience can be used as 'validated treatment criteria of metritis'. A possibility to circumvent this gap of herd specific knowledge is to perform within-herd clinical trials as proposed by Kristensen [18].

### Has the veterinary paradigm shifted in the minds of veterinarians in practice?

Herd health programmes often aim at close monitoring of disease incidence to allow timely diagnosis, subsequent intervention and evaluation of effects indicating the paradigm shift in veterinary dairy medicine from cows to herds and from treatment to prevention [19]. The results of the present study illustrate how difficult it can be to integrate a systematic approach to clinical examinations and provide useful data - even within the framework of a herd health programme. Some of the veterinarians involved in this study seemed to base both cow-level decisions and, to some extent, farm advice on personal judgements and tacit knowledge, despite their proclaimed intentions to base their daily practice to a higher degree on epidemiological considerations. The results of this study indicate that it is difficult to obtain valid data across herds and between veterinarians when their decision making procedures and motivation to collect data are so different.

## Conclusion

Variation and bias in data based on clinical examinations can be linked to veterinarians' individual perception of the purpose of, and their motivations for, data collection. Some veterinarians conduct clinical examinations to support their treatment decision, while others see it as either as a data collection scheme for use at herd level or national level. A model of understanding is developed based on veterinarians' considerations and procedures involving both individual cow characteristics and factors at farm and population level. The study demonstrates that treatment decisions often are likely to be based on both implicit and explicit types of information. Factors identified in the study were the individual cow's general clinical condition and anamnesis, herd and farm related factors, common treatment strategies developed in groups of veterinarians, as well as the veterinarian's perception of the prognosis for treatment(s) with regard to production, economy, animal health and welfare. Acknowledgement of the interaction between human decisions, motivations for disease recording and data quality can potentially lead to improved data quality and/or improved interpretations of the results of quantitative data analyses if the knowledge is communicated to both practicing veterinarians and educational systems. The identified sources of variation and bias should be taken into consideration by researchers and decision makers (e.g. in organisations and governmental institutions).

## Competing interests

The authors declare that they have no competing interests.

## Authors' contributions

DBL has conceptualized and conducted the interviews, transcribed and performed the major parts of the analysis and writing process. MV has contributed substantially with regard to the methodology, analysis and writing. CE has revised the manuscript critically for important intellectual content, in addition to his contribution to the general concepts of the study. All authors has read and approved on the contents of the final manuscript
